# Supplementary material for: Asynchronous magnetic resonance elastography: Shear wave speed reconstruction using noise correlation of incoherent waves
Source: Magn Reson Med. 2022 Oct 27;89(3):990–1001. doi: 10.1002/mrm.29502 (PMC9792433; doi:10.1002/mrm.29502)
Supplement: Supplementary file 1 — DATA S1 MATLAB code used to generate the numerical simulations of Figure 1 [file MRM-89-990-s002.zip › k-Wave/helpfiles/k-wave_photoacoustic_image_reconstruction.html]

Photoacoustic Image Reconstruction (k-Wave)


# Photoacoustic Image Reconstruction

- Example: 2D FFT Reconstruction For A Line Sensor
- Example: 3D FFT Reconstruction For A Planar Sensor
- Example: 2D Time Reversal For A Line Sensor
- Example: 2D Time Reversal For A Circular Sensor
- Example: 3D Time Reversal For A Planar Sensor
- Example: 3D Time Reversal For A Spherical Sensor
- Example: Image Reconstruction With Directional Sensors
- Example: Image Reconstruction With Bandlimited Sensors
- Example: Attenuation Compensation Using Time Reversal
- Example: Attenuation Compensation Using Time Variant Filtering
- Example: Automatic Sound Speed Selection
- Example: Iterative Image Improvement Using Time Reversal
- Example: Iterative Image Reconstruction Using The Adjoint
